# PTEN ameliorates autoimmune arthritis through down-regulating STAT3 activation with reciprocal balance of Th17 and Tregs

**DOI:** 10.1038/srep34617

**Published:** 2016-10-06

**Authors:** Seung Hoon Lee, Jin-Sil Park, Jae-Kyung Byun, JooYeon Jhun, KyungAh Jung, Hyeon-Beom Seo, Young-Mee Moon, Ho-Youn Kim, Sung-Hwan Park, Mi-La Cho

**Affiliations:** 1 The Rheumatism Research Center, Catholic Research Institute of Medical Science, The Catholic University of Korea, Seoul, South Korea; 2 Impact Biotech, Korea 505 Banpo-Dong, Seocho-Ku, 137-040, Seoul, Korea; 3 Division of Rheumatology, Department of Internal Medicine, School of Medicine, The Catholic University of Korea, Seoul, Republic of Korea; 4 Laboratory of Immune Network, Conversant Research Consortium in Immunologic disease, College of Medicine, The Catholic University of Korea, Seoul, South Korea

## Abstract

PTEN is a tyrosine phosphatase with significant function in inhibiting STAT3 activation. Recently, inactivation of STAT3 has been demonstrated as a therapeutic candidate for autoimmune arthritis. The expression of PTEN controlled by p53 regulates autoimmune arthritis through modulating the balance between Th17 and Treg. We hypothesized that PTEN regulated by p53 might reduce CIA severity and inflammatory response via inhibiting STAT3 activation. Our results revealed that PTEN could ameliorate experimental autoimmune arthritis by reducing STAT3 activity and Th17 differentiation. Systemic infusion of PTEN overexpression downregulated CIA severity. In addition, PTEN overexpression decreased the activation of T cells and modulated reciprocal differentiation of Th17 and Treg cells. We observed that PTEN expression downregulated by p53 deficiency induced the activation of STAT3. Loss of p53 exacerbated autoimmune arthritis and dysregulated the population of Th17 and Treg. These data suggest that induction of STAT3-modulatory activity of PTEN may be a therapeutic target for rheumatoid arthritis therapy.

Rheumatoid arthritis (RA) is a complex autoimmune disorder that induces chronic inflammatory response. Although RA pathogenesis is not apparent, effector T cell differentiation is involved because RA is characterized by vast inflammation. It has been well documented that IL-17 secreted by T helper (Th) 17 cells is enhanced in peripheral blood of RA patients compared to that of normal subjects^1^. Moreover, several proinflammatory cytokines are associated with the augmentation of RA^2^. Especially, IL-17 expression can lead to chronic immune inflammatory response in patients with RA^3^. IL-17 production is also upregulated in RA patients compared to that in healthy controls^4^.

Signal transducer and activator of transcription (STAT) 3, a member of DNA binding transcription factor, performs a significant role in inflammation by modulating various cytokines production and T cell lineage. For example, STAT3 activation promotes IL-17 level^5^,^6^. Inflammatory CD4^+^ T cells such as Th17 are upregulated by STAT3^6^,^7^. In addition, STAT3 directly controls Th17 differentiation as a transcription factor^8^. STAT3 plays a key role in immune inflammatory response. It is a potential target for treatment of RA. It has been suggested that STAT3 inhibition can attenuate experimental autoimmune arthritis progression and downregulate Th17 differentiation^9^,^10^.

Phosphatase and tensin homolog (PTEN), a tumor suppressive factor, is a 3′-specific phosphatidylinositol 3,4,5-treiphosphate phosphatase^11^. This protein is also involved in RA. Indeed, downregulated PTEN expression is a characteristic of activated synovial fibroblast of RA patients^12^. It has been well documented that PTEN can downregulate STAT3 activation^13^. Moreover, PTEN plays a key role in the development of immune response. Recently, it has been revealed that PTEN can increase Treg stability and that the loss of PTEN can lead to spontaneous inflammatory disorder^14^. PTEN production is associated with tumor protein p53 involving in the reduction of autoimmune inflammatory response^15^. It has been demonstrated that transcription of PTEN is controlled by p53^16^.

We hypothesized that PTEN could attenuate the development of autoimmune arthritis by reducing STAT3 activation and Th17 cells differentiation. Previously, we have reported that p53 could control autoimmune arthritis through STAT3 mediated balance between Th17 and Treg^17^. The present study was conducted to identify whether PTEN had therapeutic potential related to p53 in autoimmune arthritis. Thus, we evaluated the therapeutic efficacy of PTEN in experimental autoimmune arthritis.

## Results

### Overexpression of PTEN ameliorates CIA development

**Figure 1:**
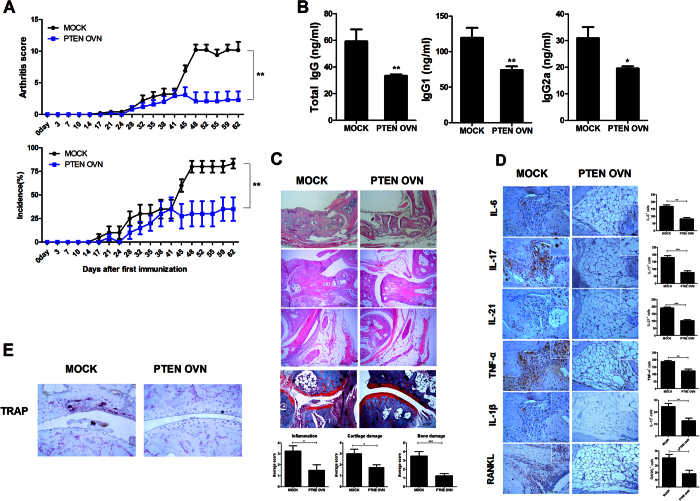
PTEN ameliorates CIA development. PTEN or mock vector was administered systemically one time in a week into CIA induced mice. Mice were sacrificed ot 9 weeks after first immunization. (**A**) Clinical scores in CIA induced mice (*P < 0.05, n = 10). (**B**) The levels of IgG, IgG1, and IgG2a antibodies in each group. Data are presented as mean ± SD of three independent experiments (*P < 0.05, **P < 0.03, n = 10). (**C**) Joint tissues from CIA mice stained by H&E (original magnification, 40× or 200×, n = 6) or safranin O (original magnification, 200×, n = 6). (**D**,**E**) Immunohistochemical detection of IL-6, -21, -17, IL-1β, TNF-α, RANKL, and TRAP after staining in the synovium of CIA mice. (Original magnification, 200×, n = 6). All histological analyses were performed at least 3 times. Representative images are revealed. Data are presented as mean ± SD of three independent experiments (*P < 0.05).

To determine whether PTEN had anti-arthritic effect, CIA induced mice were injected with either PTEN overexpression or mock vector once weekly. PTEN overexpression significantly downregulated the severity of arthritis in CIA induced mice (Fig. 1A). The concentrations of total IgG, IgG1, and IgG2a in the serum were significantly decreased in mice injected with PTEN overexpression compared to mock group (Fig. 1B). PTEN overexpression significantly reduced the degree of inflammation, bone damage, and cartilage damage (Fig. 1C). Immunohistochemical analysis revealed that injection with PTEN overexpression vector significantly suppressed the expression of proinflammatory cytokines and osteoclastogenesis related factor such as RANKL and TRAP in joints compared to CIA mice treated with mock vector (Fig. 1D,E). Our results suggested that PTEN overexpression could suppress CIA severity, thus reducing inflammatory response and osteoclastogenesis in joint.

### Overexpression of PTEN regulates reciprocal Th17/Treg balance in CIA

**Figure 2:**
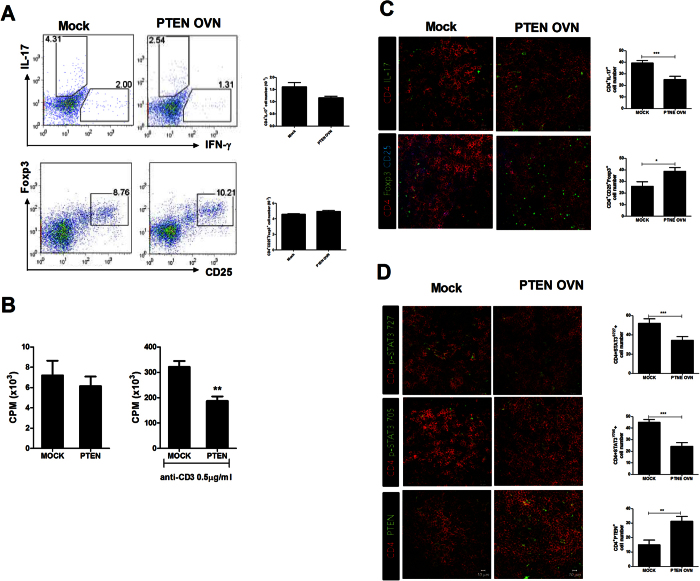
PTEN regulates reciprocal Th17/Treg balance in CIA. PTEN overexpression enhances Treg cells and decreases p-STAT3 expression in the CD4^+^ T cells in CIA mice. (**A**) The populations of IFN-γ, IL-17 and Foxp3 producing CD4^+^ T cells were analyzed by intracellular flow cytometric analysis. (**B**) T cell activity of LBRM transfected by PTEN or mock vector was assessed by T cell proliferative respond assay. (**C**) Spleens of CIA mice were subjected to immunostaining for CD4^+^IL-17 or CD4^+^CD25^+^Foxp3^+^ cells. (Original magnification, 40×) (**D**) Spleens of CIA mice were subjected to confocal staining for CD4^+^pSTAT3y705^+^ or CD4^+^pSTAT3s727^+^ cells (original magnification, 40×). The number of cells was counted in four independent quadrants. Data are presented as mean ± SD of three independent experiments (*P < 0.05, ***P < 0.01, n = 6).

To determine whether PTEN overexpression could attenuate dysregulated balance between Th17 and Treg, we examined the differentiation of Th17 and Treg in CIA mice. The overexpression of PTEN reduced Th17 differentiation in the spleen tissues of CIA induced mice. However, the differentiation of Treg cell was promoted in CIA induced mice injected with PTEN overexpression vector (Fig. 2A). It is well documented that T cell activation is involved in the pathogenesis of RA^18^. In mixed lymphocyte reaction (MLR), the alloreactive T cell response was decreased in LBRM transfected with PTEN overexpression compared to that in the control (Fig. 2B). Additionally, PTEN overexpression decreased the number of IL-17 producing CD4^+^p-STAT705^+^ or p-STAT727^+^ T cells. However, the number of CD4^+^p-Foxp3^+^ T cells in the spleen tissues of CIA induced mice was significantly upregulated compared to that in the spleen tissues of mice treated with mock vector based on immunofluorescence confocal microscopy (Fig. 2C,D). These data suggested that PTEN overexpression ameliorated the imbalance between Th17 and Treg in CIA.

### Loss of p53 induces STAT3 activation

**Figure 3:**
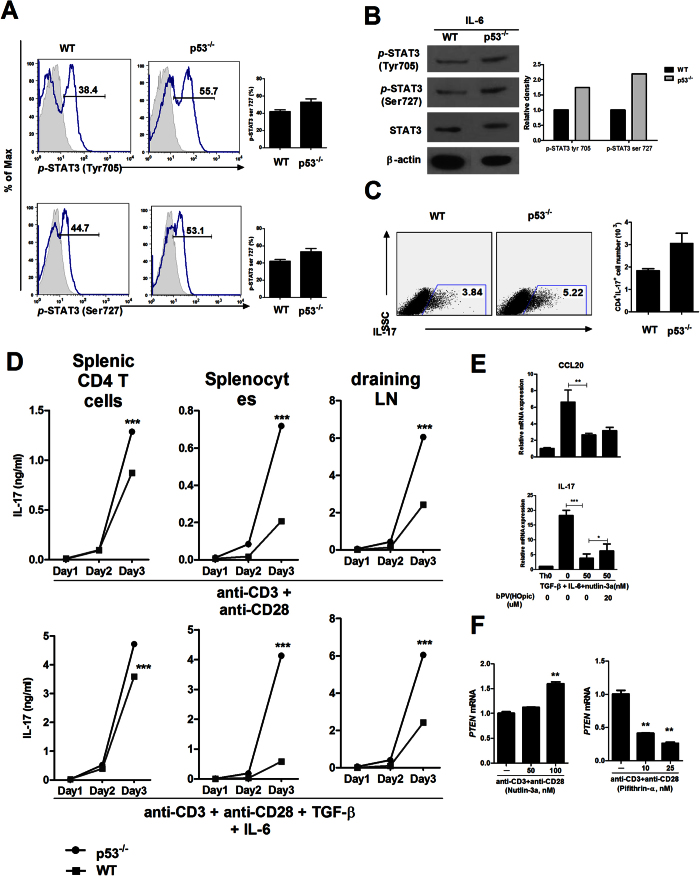
p53 deficiency induces STAT3 activation. (**A**,**B**) The expression levels of p-STAT3 Tyr705 and p-STAT3 Ser727 in splenocytes from WT and p53^−/−^ mice stimulated by IL-6 (20 ng/ml) for 1 hour were determined by flow cytometry and western blot. (**C**,**D**) IL-17A production in splenic CD4^+^ T cells, splenocytes, and draining lymph nodes from WT and p53^−/−^ mice after incubation for 3 days under Th0 cell conditions (stimulation only with anti-CD3 and anti-CD28 without added cytokines) or Th17 cell–polarizing conditions (stimulation only with anti-CD3 and anti-CD28 with TGF-β and IL-6) were examined by ELISA and flow cytometry. (**E**) Relative mRNA expression levels of IL-17 and CCL20 in mice splenocytes after incubation for 1 day under Th0 cell conditions and treated with nutlin-3a or bPV(HOpic) were determined by real-time PCR. (**F**) Relative mRNA expression levels of PTEN in mice splenocytes treated with nutlin-3a or pifithrin-α were determined by real-time PCR. Data are presented as mean ± SD of three independent experiments (**P < 0.03, ***P < 0.01).

Previously, p53 has been known as a modulator of STAT3 activation through significant reducing STAT3 phosphorylation and STAT3 DNA binding activity^19^. The phosphorylation levels of STAT3 Tyr705 and Ser727 in p53 deficient mice splenocytes were increased compared to those of WT mice splenocytes (Fig. 3A,B). Cells isolated from WT or p53 deficient mice were cultured under the condition of Th0 or Th17. IL-17 production in p53^−/−^ mice was significantly increased compared to that in WT mice (Fig. 3C,D). Gene expression of IL-17 and CCL20 which causes recruiting of IL–17 expressing cells^20^ in WT mice splenocytes was increased after stimulation with TGF-β and IL-6. But, mRNA expression of IL-17 and CCL20 was reduced significantly by p53 activator, nutlin-3a. In contrast, bPV(HOpic), the inhibitor of PTEN, promoted gene expression of IL-17 and CCL20 (Fig. 3E). Gene expression of PTEN was enhanced significantly by nutlin-3a. However, pifithrin-α, the inhibitor of p53, significantly decreased the mRNA level of PTEN in mice splenocytes (Fig. 3F). These results suggested that the inhibition of p53 under inflammatory milieu of RA might have enhanced inflammation. Additionally, PTEN expression could be regulated by p53 dependent manner.

### Loss of p53 exacerbates induction of Th17 and reduction of Treg

**Figure 4:**
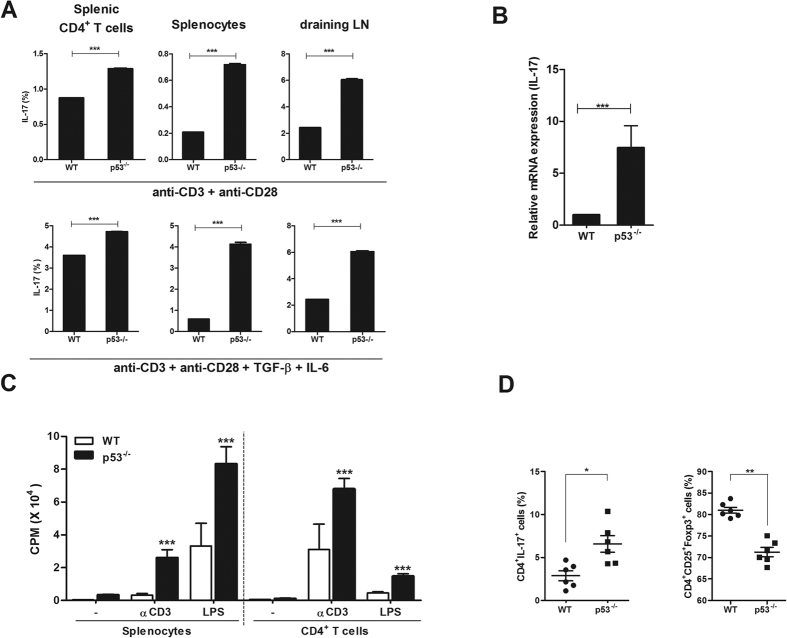
p53 deficiency exacerbates the induction of Th17 and the reduction of Treg. (**A**) IL-17A production in splenic CD4^+^ T cells, splenocytes, and draining lymph nodes from WT and p53^−/−^ mice after incubation for 3 days under Th0 cell conditions (stimulation only with anti-CD3 and anti-CD28 without added cytokines) or Th17 cell–polarizing conditions (stimulation only with anti-CD3 and anti-CD28 with TGF-β and IL-6) was examined by flow cytometry. (**B**) Relative mRNA levels of IL-17 in splenic CD4^+^ T cells from WT and p53^−/−^ mice were determined by real-time PCR. (**C**) T cell activity of in splenic CD4^+^ T cells and splenocytes from WT and p53^−/−^ mice was assessed by T cell proliferative respond assay. (**D**) The populations of IL-17, CD25, and Foxp3 expressing CD4^+^ T cells were analyzed by intracellular flow cytometry. Data are presented as mean ± SD of three independent experiments (**P < 0.03).

Recently, p53 has been demonstrated as a mediator of balance between Th17 and Treg in RA^17^. Cells of WT or p53 deficient mice in normal state were cultured under Th0 or Th17 condition. The expression of IL-17 was significantly increased (Fig. 4A). Gene expression of IL-17 in splenocytes isolated from p53 deficient mice was also significantly increased compare to that in WT mice (Fig. 4B). T cell activation was profoundly upregulated in cells isolated from p53 deficient mice compared to that in WT mice (Fig. 4C). Th17 differentiation in p53 deficient mice was significantly increased whereas Treg differentiation was significantly reduced compared to that in WT mice (Fig. 4D). These data suggested that p53 deficiency could lead to T cell activation and imbalance between Th17 and Treg.

### p53 deficiency exacerbates CIA severity by upregulating inflammation

**Figure 5:**
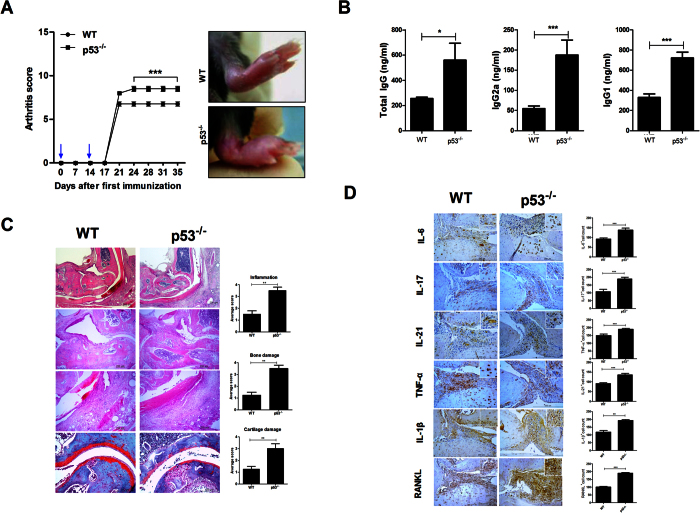
p53 deficiency exacerbates CIA severity. Mice were sacrificed on day 35 after the first immunization. (**A**) Clinical scores ankle condition in CIA induced WT and p53^−/−^ mice (***P < 0.05, n = 10). (**B**) The expression levels of IgG, IgG1, and IgG2a antibodies in each group were examined. Data are presented as mean ± SD of three independent experiments (*P < 0.05, ***P < 0.01, n = 10). (**C**) Joint tissues from CIA induced WT and p53^−/−^ mice after staining with H&E (original magnification, 40× or 200×, n = 6) or safranin O (original magnification, 200×, n = 6). (**D**) Immunohistochemical detection of IL-6, IL-21, IL-17, IL-1β, TNF-α, and RANKL in the synovium of CIA induced WT or p53^−/−^ mice after staining (original magnification, 200×, n = 6). All histological analyses were performed at least 3 times. Representative images are revealed. Data are presented as mean ± SD of three independent experiments (**P < 0.03, ***P < 0.01).

Based on arthritis scores, it was found that p53 deficiency exacerbated CIA progression *in vivo* (Fig. 5A). Serum levels of total IgG, IgG1, and IgG2a were significantly increased in p53 deficiency mice compared to those in WT mice (Fig. 5B). Moreover, histological analysis showed that paws and ankles of p53 deficiency arthritis mice had higher degree of inflammation with bone damage and cartilage damage (Fig. 5C). The expression of proinflammatory cytokines in joints were significantly upregulated in p53 deficiency arthritis mice compared to that in WT mice (Fig. 5D). Our results suggested that p53 deficiency might have failed to regulate inflammatory response, thus worsening local inflammatory milieu.

### p53 deficiency decreases PTEN expression and induces imbalance between Th17 and Treg

**Figure 6:**
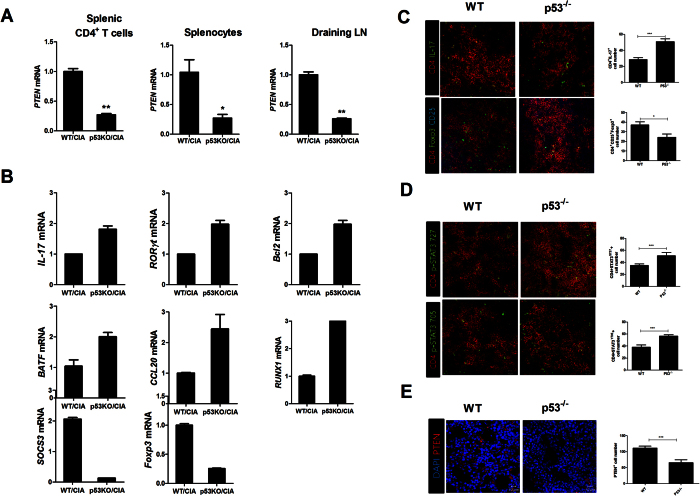
p53 deficiency decreases PTEN expression and induces imbalance between Th17 and Treg. (**A**,**B**) Relative mRNA levels of PTEN and factors such as IL-17, RORγt, and Foxp3 involved in the differentiation of Th17 and Treg in splenic CD4^+^ T cells, splenocytes, and draining lymph nodes from CIA induced WT or p53^−/−^ mice were assessed by real-time PCR. Data are presented as mean ± SD of three independent experiments (*P < 0.05, **P < 0.03). (**C**) Spleens of CIA mice were subjected to immunostaining for CD4^+^IL-17 or CD4^+^CD25^+^Foxp3^+^ cells. (**D**) Spleens of CIA mice were subjected to confocal staining for CD4^+^pSTAT3y705^+^ or CD4^+^pSTAT3s727^+^ cells. The number of cells was counted in four independent quadrants. Data are presented as mean ± SD of three independent experiments (*P < 0.05, ***P < 0.01, n = 6).

Since PTEN expression is regulated by p53^16^, we investigated PTEN expression in p53 deficiency mice with CIA. Gene expression levels of PTEN in splenic CD4^+^ T cells, splenocytes, and draining lymph nodes isolated from p53 deficiency mice with CIA were decreased significantly compared to those in WT mice with CIA (Fig. 6A). Since RA can result in dysregulated balance of Th17/Treg^1^, we also measured the balance between Th17 and Treg. The mRNA level of Th17 related molecules including IL-17 was increased, whereas the mRNA expression of Treg cell-related molecules such as Foxp3 was enhanced in p53 deficiency mice with CIA (Fig. 6B). In addition, the number of CD4^+^p-STAT5^+^ T cells in the spleen tissues of p53 deficiency mice with CIA was significantly downregulated compared to that in the spleen tissues of WT mice with CIA based on immunofluorescence confocal microscopy (Fig. 6C). However, loss of p53 promoted the number of IL-17 producing CD4^+^p-STAT705^+^ or p-STAT727^+^ T cells in the spleen tissues based on immunofluorescence confocal microscopy (Fig. 6D). Moreover, PTEN expression was significantly downregulated in p53 deficient mice compared to that in WT mice (Fig. 6E). These results demonstrated that p53 deficiency could accelerate the imbalance between Th17 and Treg in CIA.

## Discussion

Until now, PTEN has been investigated extensively as a tumor suppressor with a role in cell metabolism, motility, and tumor microenvironment^21^. Recently, PTEN has been observed to be associated with cell differentiation as a phosphatase^22^. In addition, PTEN is revealed to have therapeutic effect in rat with CIA^23^. However, little is known about the process of PTEN function associated with p53 in RA. Here, we studied the therapeutic activity of PTEN in RA and identified a new mechanism of RA regulation.

The most notable observation of this investigation is that PTEN can attenuate RA via reciprocal differentiation of Th17/Treg. To our knowledge, this is the first research to provide evidence that PTEN could be used for RA therapy through regulating Th17/Treg balance. Previously, a number of documents have demonstrated that imbalance between Th17 and Treg can contribute to RA^1^,^24^. Modulation of Th17 and Treg cells has an important role in RA therapy^10^,^25^. The activation of STAT5 prolongs Foxp3 production in Treg, while STAT3 activation increases the differentiation of Th17 cells^26^. Moreover, binding of p-STAT3 and p-STAT5 can competitively regulate IL-17 transcription^27^. Our results demonstrated that Foxp3^+^ T cells were induced by PTEN overexpression while p53 deficiency significantly induced p-STAT3^+^ T cells. This could be due to the diminishment of T cell transcriptional regulators such as Foxp3 and SOCS3 and the enhancement of RORγt, RUNX1, and BATF. In addition, PTEN decreased the number of Treg cells while Th17 differentiation was promoted in RA mice model. Hence, PTEN might be another efficient mechanism regulated by which p53 through controlling Th17/Treg balance.

Tumor protein 53 (p53), a tumor suppressor factor, is essential for cellular response to DNA damage. It plays a key role in several gene expression as a resourceful transcription factor under stressful conditions. It has been demonstrated that p53 is involved in a variety of cellular signal pathways, cell proliferation, and apoptosis^28^,^29^,^30^. Although p53 function has been recognized mainly in the cell cycle, emerging evidence has suggested that p53 has important role not only in apoptosis, cell differentiation, and DNA repair, but also in the modulation of STAT-mediated Th17 cells^17^. Loss of p53 can promote the progression of antigen-induced arthritis and increase activated T cell differentiation^31^. In this study, PTEN expression was found to be regulated in p53 dependent manner. Additionally, p53 deficiency aggravated CIA severity and reduced PTEN expression. These results suggested that PTEN could have therapeutic effect in autoimmune arthritis through p53.

IL-17 is a typical proinflammatory cytokine inducing the expression of IL-6, -21 and TNF-α^32^,^33^,^34^. It has been documented that IL-17 can upregulate proinflammatory cytokines including IL-6 and IL-8 and aggravate joint inflammation of RA through activating CD4^+^ T cells^35^,^36^. Th17 secreting IL-17 performs a key role in the pathogenesis of RA. Th17 frequency and IL-17 level are strikingly correlated with RA development. It has been documented that Th17 can result in excessive inflammation in patients with RA^3^. Our data revealed that PTEN overexpression reduced the activation of T cells and that the loss of p53 enhanced the proliferation of Th17 cells. Since IL-17 expression is well known to induce RA development, suggesting a novel therapeutic strategy to modulate RA via PTEN expression.

Granulocyte-macrophage colony-stimulating factor (GM-CSF), an immune modulatory cytokine, performs a significant role in immune tolerance and attenuates autoimmune disorder^37^. It has been suggested that GM-CSF suppressed progression of autoimmune disease via induction of Tregs^38^. Previously, immune tolerance can be a good strategy for CIA therapy. Indeed, immune tolerance induction using CII showed therapeutic effect *in vivo* and *in vitro*^39^. CD8^+^ Tregs can reveal therapeutic implications in CII involved-disease inducing immune tolerance^40^. The therapeutic effect of PTEN in CIA may be involved in upregulation of GM-CSF and immune tolerance. Thus, further study will be needed to confirm therapeutic activity of PTEN related with GM-CSF expression and immune tolerance.

We analyzed the gene expression of p53 and STAT3 in CD4^+^ T cells of healthy individuals and RA patients from the National Center for Biotechnology Information Gene Expression Omnibus database (GSE4588). The GSE4588 database contains 10 healthy subjects and 8 RA patients with clinic and pathological information. We observed that the relative mRNA level of STAT3 of RA patients was promoted significantly compared to that of healthy individuals in this database. The mRNA expression of p53 in RA patients was downregulated significantly compared to that in healthy individuals. The lack of p53 might have aggravated RA progression and induced STAT3 activation. In this study, we demonstrated that PTEN overexpression could reduce CIA progression and that the loss of p53 enhanced the expression of STAT3.

The function of PTEN and p53 in STAT3 activation has already been studied in previous investigations^13^,^19^. Recently, p53 deficiency has revealed correlation with RA severity inducing Th17 differentiation^17^. However, our study demonstrated significant mechanism of PTEN associated with p53 in the development of CIA via reciprocally regulating Th17 and Treg. This preliminary evidence suggested that upregulating PTEN could be a strong therapeutic strategy for the treatment of RA.

## Materials and Methods

### Ethics statement

The Animal Care Committee of The Catholic University of Korea approved the experimental protocol, and all the experimental procedures were carried out in accordance with the protocols approved by the Animal Research Ethics Committee at the Catholic University of Korea. All procedures performed followed the ethical guidelines for animal studies.

### Animals

Male DBA1/J mice and C57BL/6 mice at 6–8 weeks old (Orient, Korea) were maintained in groups of five in polycarbonate cages in a specific pathogen-free environment. They were provided free access to standard mouse chow (Ralston Purina, Gray Summit, MO) and water *ad libitum*. Mice harboring the p53-null allele with a C57BL/6 mice background (B6.129S2-Trp53tm1Tyj/J) were obtained from The Jackson Laboratory.

### Induction of arthritis and injection of agents

Collagen-induced arthritis (CIA) was induced in DBA1/J mice (each group: n = 10). Mice were immunized with 100 μg of chicken CII (Chondrex Inc., Redmond, WA, USA) dissolved overnight in 0.1N acetic acid (4 mg/ml) in complete Freund’s adjuvant or incomplete Freund’s adjuvant (Chondrex Inc). The immunization was performed intradermally into the base of the tail. CIA was induced in the p53^−/−^ strain mice as described previously^41^. Eight days after immunization, mice were injected intravenously with 100 μg of PTEN or mock vector in 2 ml of saline over a 10-second period. After 8 days, the same mice received intramuscular injection of 50 μg of PTEN or mock vector in the left leg with electrical stimulation (electroporation) using a 31-gauge needle insulin syringe for hydrodynamic-based procedures. Two days later, mice received an intramuscular injection of 50 μg of PTEN or mock in the right leg through electroporation.

### Clinical scoring and histological assessment of arthritis

Arthritis score was measured visually twice per week based on the appearance of arthritis in the joints and graded according to Williams *et al*.^42^. The joints of each mouse were fixed in 10% formalin, decalcified in 10% EDTA, and embedded in paraffin wax for histological analysis. Hematoxylin-eosin (H&E) stained sections were scored for inflammation, destruction of cartilage, and bone damage according to published criteria^43^,^44^.

### Real-time polymerase chain reaction (PCR)

Total RNA was isolated using TRI Reagent (Molecular Research Center, Inc. Cincinnati, OH, USA) according to the manufacturer’s instructions. Complementary DNA was synthesized using SuperScript Reverse Transcription system (Takara). A Light-Cycler 2.0 instrument (software version 4.0; Roche Diagnostics) was used for PCR amplification. All reactions were performed using LightCycler FastStart DNA Master SYBR Green I mix (Takara) following the manufacturer instructions. Primer sequences used to amplify mouse genes are listed in Supplementary Table 1.

### Flow cytometry

Flow cytometry was conducted as described previously^45^,^46^. Cells were immunostained with various combinations of fluorescent antibodies against CD4, CD25, FOXP3, IFN-γ, IL-4, and IL-17 (eBioscience, San Diego, CA, USA). Prior to intracellular staining, cells were restimulated with phorbol myristate acetate (PMA; 25 ng/mL) and ionomycin (250 ng/mL) for 4 hours in the presence of GolgiSTOP (BD Biosciences). For analysis of Treg cells, cells were surface labeled with CD4 and CD25, followed by fixation, permeabilization and intracellular staining with Foxp3 was perfirmed per the manufaturer’s protocol. Flow cytometry was performed on a FACSCalibur flow cytometer (BD Biosciences). The data was analyzed using the FlowJo software (Tree Star, Ashland, OR,USA).

### ELISA

Enzyme-linked immunosorbent assay (ELISA) was conducted as described previously^47^,^48^. Briefly, blood was obtained from the orbital sinus of mice. Serum levels of IgG antibodies were measured using a commercially available ELISA kit (Bethyl Laboratories, Montgomery, TX, USA). Horseradish peroxidase (HRP) activity was measured using tetramethyl benzidine as substrate (eBioscience, San Diego, CA, USA).

### Staining for confocal microscopy analysis

Tissue cryosections (7 μm thick) were fixed with acetone and stained with FITC-, PE-, PerCP-Cy5.5-, or APC-conjugated monoclonal antibodies against mouse CD4, pSTAT3 (Tyr 705, Ser 727), pSTAT5, IL-17, and FOXP3 (eBioscience). After incubation at 4 °C overnight, stained sections were visualized through confocal microscopy (LSM 510 Meta; Zeiss, Göttingen, Germany).

### Immunohistochemistry

Immunohistochemistry was performed using the Vectastain ABC kit. Tissues were first incubated with primary anti-c-Jun and anti-c-Fos antibodies overnight at 4 °C. The primary antibody was detected with a biotinylated secondary antibody followed by incubation with a streptavidin-peroxidase complex for 1 h. DAB chromogen was added to obtain colored product.

### Transfection

PTEN vector purchased from Addgene (plasmid#22231) was used to generate the overexpression of PTEN. Mock and PTEN vector constructs were transfected into LBRM (mice T lymphoma cell line) cells using Amaxa 4D-Nucleofector X unit according to the manufacturer’s recommendations with program DN-100 (Lonza).

### Murine T cell isolation and alloreactive T cell responses *in vitro*

Splenocytes were harvested in ACK lysis buffer, washed, and resuspended in complete culture medium (RPMI 1640 supplemented with 10% [v/v] heat-inactivated fetal calf serum). To purify splenic CD4^+^ T cells, splenocytes were incubated with anti-CD4-coated magnetic beads, and CD4^+^ T cells were isolated using magnetic-activated cell sorting (MACS) separation columns (Miltenyi Biotec). The cells were pretreated with Pifithrin-α, Nutlin-3a (Cayman Chemical) or bPV(HOpic) (Santa Cruz Biotechnology) and then stimulated under the required polarizing conditions. Aliquots of 2 × 10^5^ CD4^+^ T cells (responders) were cultured with 2 × 10^5^ irradiated (2,500 cGy) APCs in 96-well plates containing 200 μl/well of complete medium, at 37 °C in a humidified 5% (v/v) CO_2_/air atmosphere. Cells were pulsed with 1 μCi of tritiated thymidine (^3^[H]-TdR; NEN Life Science Products Inc., Boston, MA, USA) 18 h before harvesting, and counted with an automated harvester (PHD Cell Harvester; Cambridge Technology, Inc., Cambridge, MA, USA). Results are expressed as the mean cpm values of triplicate samples.

### Western blot

Western blot was performed as described previously^48^,^49^. Proteins were loaded onto 10% polyacrylamide gels and subjected to sodium dodecyl sulfate polyacrylamide gel electrophoresis followed by transferring to nitrocellulose membranes (Invitrogen Life Technologies, Carlsbad, CA, USA). Membranes were blocked with 5% (w/v) non-fat milk in Tris-buffered saline containing 0.1% Tween-20 for 1 h followed by incubation with antibodies against p-STAT3 Y705, p-STAT3 S727, t-STAT3 (Cell signaling), and β-actin (Santa Cruz Biotechnology) overnight at 4 °C. Immunoreactivity was determined using enhanced chemiluminescence reagents (Amersham Biosciences, Piscataway, NJ, USA).

### Statistical analysis

Data were presented as means ± standard deviations (SD). Statistical analysis was performed with nonparametric Mann-Whitney *U* test using Graphpad Prism v.5.01. One-way analysis of variance (ANOVA) and Bonferroni’s *post hoc* test were used for multiple comparisons. Statistical significance was considered when *p* value was less than 0.05.

## Additional Information

**How to cite this article**: Lee, S. H. *et al*. PTEN ameliorates autoimmune arthritis through down-regulating STAT3 activation with reciprocal balance of Th17 and Tregs. *Sci. Rep*. **6**, 34617; doi: 10.1038/srep34617 (2016).

## Supplementary Material

Supplementary Information
