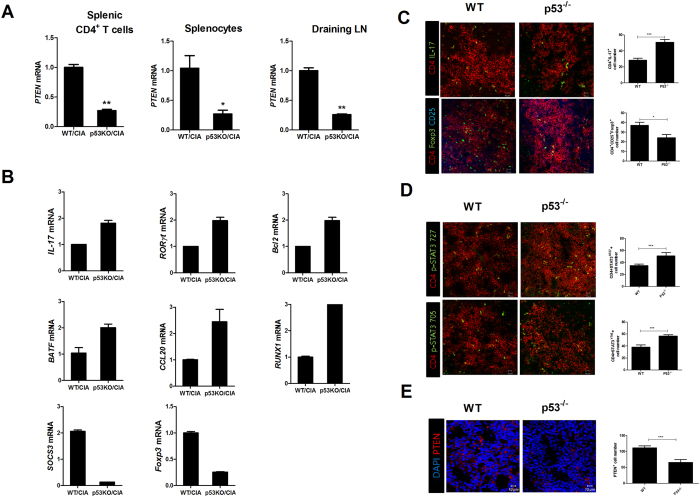# Corrigendum: PTEN ameliorates autoimmune arthritis through down-regulating STAT3 activation with reciprocal balance of Th17 and Tregs

**DOI:** 10.1038/srep42267

**Published:** 2017-09-27

**Authors:** Seung Hoon Lee, Jin-Sil Park, Jae-Kyung Byun, JooYeon Jhun, KyungAh Jung, Hyeon-Beom Seo, Young-Mee Moon, Ho-Youn Kim, Sung-Hwan Park, Mi-La Cho

Scientific Reports 6: Article number: 34617; 10.1038/srep34617 published online: 10 06 2016; updated: 09 27 2017.

This Article contains incorrect versions of Figure 3 and Figure 6. The correct Figures appear below as Figure 1 and 2 respectively.

In addition, there are typographical errors in the Methods section under the subheading ‘Staining for confocal microscopy analysis’,

“Tissue cryosections (7  μm thick) were fixed with acetone and stained with FITC-, PE-, PerCP-Cy5.5-, or APC-conjugated monoclonal antibodies against mouse CD4, pSTAT3 (Tyr 705, Ser 727), pSTAT5, IL-17, and FOXP3 (eBioscience).”

should read:

“Tissue cryosections (7 μm thick) were fixed with acetone and stained with FITC-, PE-, PerCP-Cy5.5-, or APC-conjugated monoclonal antibodies against mouse CD4, pSTAT3 (Tyr 705, Ser 727), pSTAT5, IL-17, FOXP3 and PTEN (eBioscience).”

In addition, under the subheading ‘Immunohistochemistry’,

“Tissues were first incubated with primary anti-c-Jun and anti-c-Fos antibodies overnight at 4 °C”

should read:

“Tissues were first incubated with primary anti-IL-1β, anti-IL-6, anti-IL-17, anti-IL-21, anti-TNF-α and anti-RANKL antibodies overnight at 4 °C.”

## Figures and Tables

**Figure 1:**
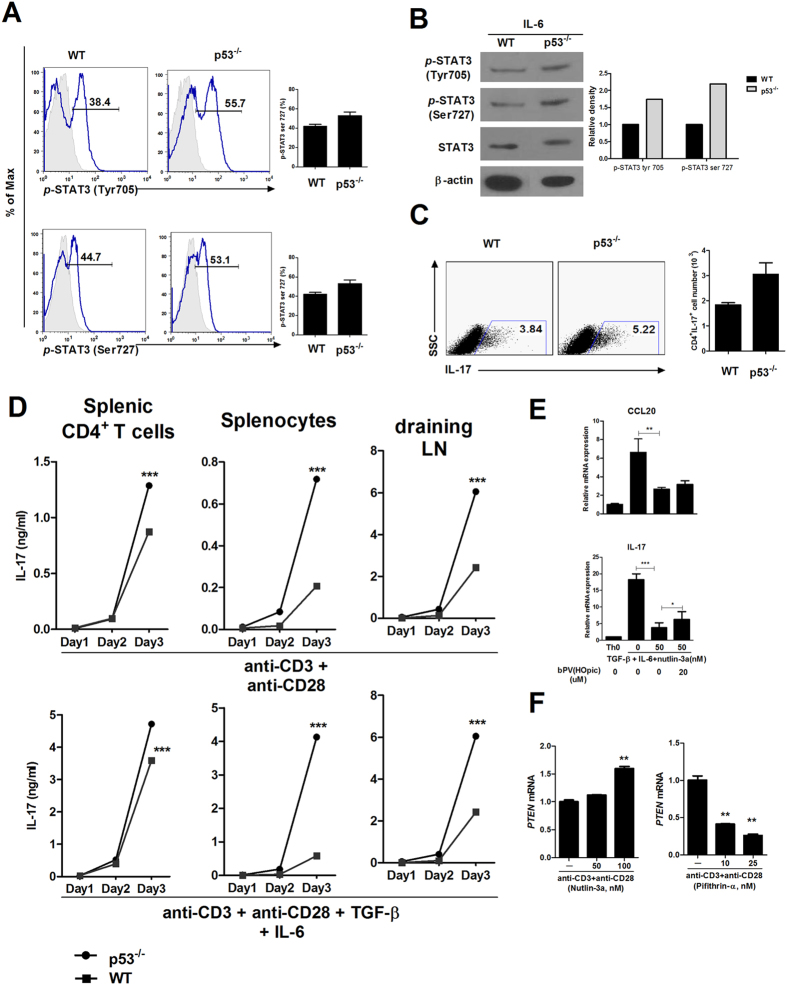


**Figure 2:**