# Supplementary material for: PTEN ameliorates autoimmune arthritis through down-regulating STAT3 activation with reciprocal balance of Th17 and Tregs
Source: Sci Rep. 2016 Oct 6;6:34617. doi: 10.1038/srep34617 (PMC5052580; doi:10.1038/srep34617)
Supplement: Supplementary Information [file srep34617-s1.pdf]

PTEN ameliorates autoimmune arthritis through down-regulating STAT3 activation with reciprocal balance of Th17 and Treg

Seung Hoon Lee[1,*], Jin-Sil Park[1, *], Jae-Kyung Byun[1], JooYeon Jhun[1], KyungAh Jung[3], Hyeon-Beom Seo[1], Young-Mee Moon[1], Ho-Youn Kim[1,4], Sung-Hwan Park[1,4, #], and Mi-La Cho[1,2, #]

[1]The Rheumatism Research Center, Catholic Research Institute of Medical Science, The Catholic University of Korea, Seoul, South Korea

[2]Laboratory of Immune Network, Conversant Research Consortium in Immunologic disease, College of Medicine, The Catholic University of Korea

[3]Impact Biotech, Korea 505 Banpo-Dong, Seocho-Ku, 137-040, Seoul, Korea

[4]Division of Rheumatology, Department of Internal Medicine, School of Medicine, The Catholic University of Korea, Seoul, Republic of Korea

Key word : PTEN, p53, Rhematoid arthritis, STAT3

[*]These authors contributed equally to this work.

[#]These authors contributed equally to this work.

Sung-Hwan Park, MD, PhD

Division of Rheumatology, Department of Internal Medicine, College of Medicine, Seoul St. Mary's Hospital, The Catholic University of Korea.

E-mail: rapark@catholic.ac.kr

Mi-La Cho, PhD

Conversant Research Consortium in Immunologic disease, College of Medicine, The Catholic University of Korea, Seoul, Korea 505 Banpo-Dong, Seocho-Ku 137-040, Seoul, Korea.

E-mail: iammila@catholic.ac.kr

Tel: +82-2-2258-7467

Fax: +82-2-599-4287

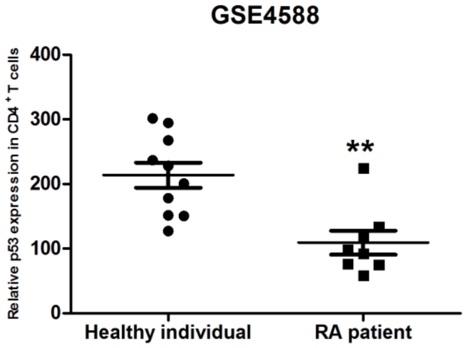

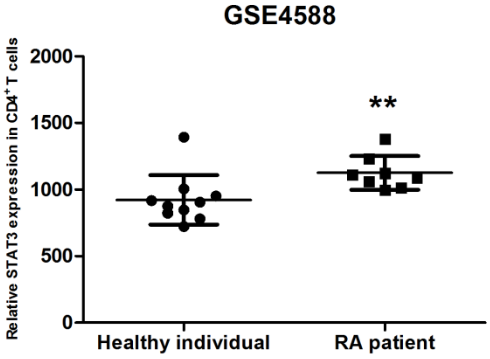

Supplementary figure 1. Gene expression of STAT3 and p53 in CD4+ T cells sorted from peripheral blood mononuclear cells of healthy individual (n = 10) or RA patients (n = 8). Data are presented as mean ± SD (*P < 0.05, **P < 0.03).

Gene	Sense primer (5'-->3)	Antisense primer (3'-->5')
PTEN	AAT TCC CAG TCA GAG GCG CTA TGT	GAT TGC AAG TTC CGC CAC TGA ACA
IL-17A	CCT CAA AGC TCA GCG TGT CC	GAG CTC ACT TTT GCG CCA AG
CCL20	CAG CTG TTG CCT CTC GTA CA	CAC CCA GTT CTG CTT TGG AT
RORγt	TGT CCT GGG CTA CCC TAC TG	GTC CAG GAG TAG GCC ACA TT
BATF	GAC AAG AAG GGC GAT GCT AC	GGC CTT AAG TCC CTC TGA CC
RUNX1	TAC CTG GGA TCC ATC ACC TC	GAC GGC AGA GTA GGG AAC TG
Foxp3	GGC CCT TCT CCA GGA CAG A	GCT GAT CAT GGC TGG GTT GT
SOCS3	CCT TTG ACA AGC GGA CTC TC	GCC AGC ATA AAA ACC CTT CA
Bcl2	GCATCCCAGCCTCCGTTAT	TGAGTACCTGAACCGGCATCT
β-actin	GAA ATC GTG CGT GAC ATC AAA G	TGT AGT TTC ATG GAT GCC ACA G

Supplementary table 1. PCR primer sequence used in this study.
